# A pixel-level coarse-to-fine image segmentation labelling algorithm

**DOI:** 10.1038/s41598-022-12532-7

**Published:** 2022-05-23

**Authors:** Jonghyeok Lee, Talha Ilyas, Hyungjun Jin, Jonghoon Lee, Okjae Won, Hyongsuk Kim, Sang Jun Lee

**Affiliations:** 1 Division of Electronics and Information Engineering, Jeonbuk National University, Jeonju-si, 54896 Republic of Korea; 2 Core Research Institute of Intelligent Robots, Jeonbuk National University, Jeonju-si, 54896 Republic of Korea; 3 Production Technology Research Division, Rural Development Administration, National Institute of Crop Science, Miryang, 50424 Republic of Korea

**Keywords:** Energy science and technology, Engineering, Mathematics and computing

## Abstract

Fine segmentation labelling tasks are time consuming and typically require a great deal of manual labor. This paper presents a novel method for efficiently creating pixel-level fine segmentation labelling that significantly reduces the amount of necessary human labor. The proposed method utilizes easily produced multiple and complementary coarse labels to build a complete fine label via supervised learning. The primary label among the coarse labels is the manual label, which is produced with simple contours or bounding boxes that roughly encompass an object. All others coarse labels are complementary and are generated automatically using existing algorithms. Fine labels can be rapidly created during the supervised learning of such coarse labels. In the experimental study, the proposed technique achieved a fine label IOU (intersection of union) of 92% in segmenting our newly constructed bean field dataset. The proposed method also achieved 95% and 92% mean IOU when tested on publicly available agricultural CVPPP and CWFID datasets, respectively. Our proposed method of segmentation also achieved a mean IOU of 81% when it was tested on our newly constructed paprika disease dataset, which includes multiple categories.

## Introduction

Artificial neural networks (ANNs) are currently utilized in a wide array of sectors, including autonomous driving, quality control, precision agriculture, smart farming, and medical image analysis systems^1–3^. Recently, deep neural networks (DNNs) have begun to be utilized to solve agricultural problems^4,5^. These neural networks are used to predict agricultural growth conditions, the presence of diseases and pests, and identify the time and type of pesticides and nutrients to be administered. ANNs can also be used to drive autonomous robots along ditches or banks^5–7^, or to categorize crops, land, weeds, fruits etc.^8–10^.

Enormous datasets must be constructed to train artificial neural networks, a process which requires a great deal of time and money. Seasons, weather, humidity, temperature, and lighting all have an impact on crops, and in images they appear to be entirely different in size, perspective, and color. Furthermore, insect damage and wild animal damage render it difficult to gather datasets. In addition, pixel-level fine labels for the pictures, once acquired, must be created. Fine labeling distinguishes the borders between crops, land, weeds, and other objects. This process requires precise disease detection in crops having overlapping boundaries with surroundings. It requires a great deal of time and effort to perform this procedure on all gathered images.

**Figure 1:**
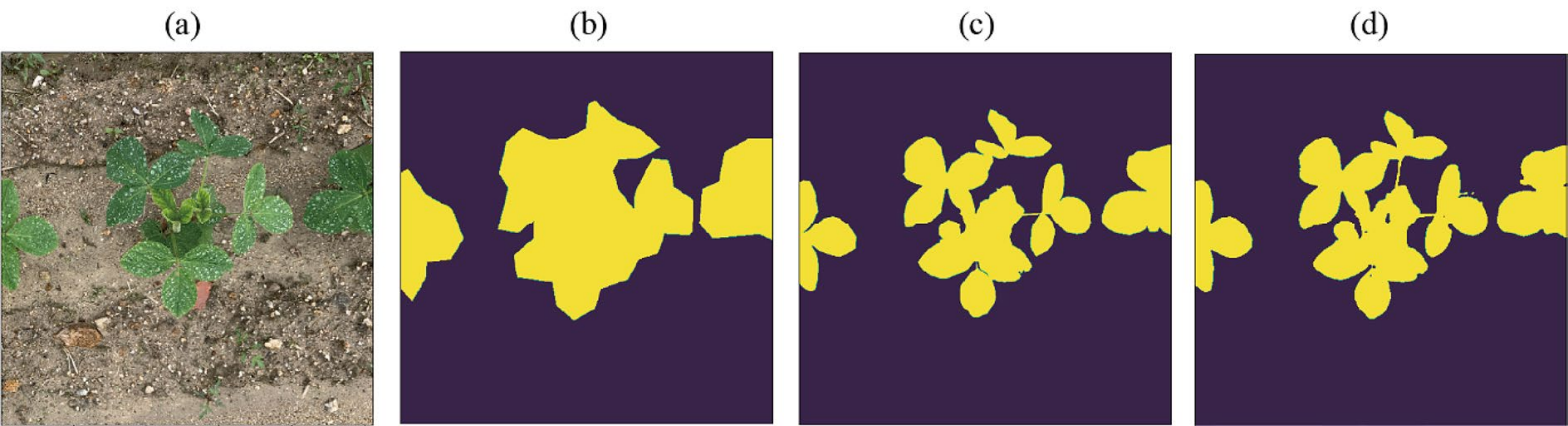
Bean-Field dataset sample. (**a**) input images, (**b**) MGRL, (**c**) CDTL, and (**d**) PLFL. Output was thresholded at 0.7.

**Figure 2:**
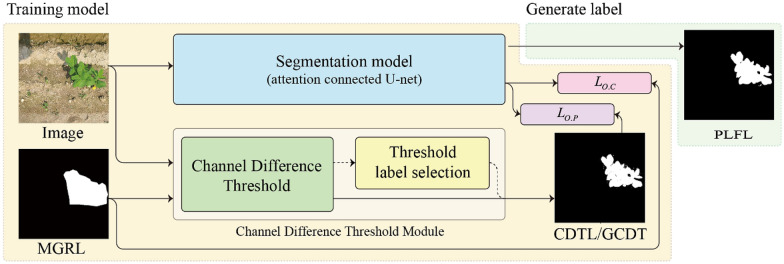
Overall framework of the proposed algorithm. The final PLFL requires a pair of pseudo labels, one generated manually and the other automatically. After that, the segmentation model can be trained with these coarse label pairs (CLPs) to generate PLFL.

In this paper, we propose a framework for the generation of pixel-level fine labels using a pair of complementary coarse labels. A single complementary coarse label pair is comprised of (1) a manual label, i.e., a manually generated rough label (MGRL) that envelopes the object of interest (Fig. 1b) and (2) a channel difference threshold label (CDTL) (Fig. 1c). A neural network that has learned the complementary labels can generate pixel-level fine labels (PLFL) (Fig. 1d) for a given dataset. The proposed method is capable of significantly reducing labelling cost and time, as it only requires manually generated rough labels (MGRL), which are easier to generate than alternatives. We demonstrate that the pixel-level fine labels (PLFL) can be generated using the proposed method in a manner that is more efficient and reliable than manual generation. Moreover, experiments show that the PLFLs generated using the proposed framework have a similarity rate of over 99% with those generated manually.

The remainder of the paper is organized as follows: first, present some recent state-of-the-art works in this field. The datasets and algorithms utilized are briefly discussed in the next section. Moving on to the following sections, we go over data configuration and validation approach, then our DNN model architecture and pre-validation hypotheses is explained. Finally, we analyze the experiments and results in detail before concluding our article with recommendations for further research.

## Related work

Machine learning applications, particularly in the deep learning domain, are rapidly diversifying and expanding^11^. To be effective, deep learning methods must employ a large amount of data for training. To date, a shortage of data has made training deep neural networks (DNNs) challenging. Further, extra data is needed to validate a trained DNN to confirm that its predictions are trustworthy^12^. Since many real-world situations do not have ideal data configurations, numerous strategies have been developed to train models effectively despite a scarcity of data.

In semi-supervised learning, a model must learn based on a limited number of labeled instances and a large number of unlabeled examples. This model must then make predictions based on new samples Generative models like the Generative Adversarial Network (GAN) and Variational Autoencoder are among the most effective semi-supervised learning methods^13–18^. Hung et al.^13^ used a fully convolutional discriminator to train the DNNs to improve segmentation masks using a combination of labeled and unlabeled data. To address the problem of inaccurate boundary detection and incorrect class assignment of large regions, Mittal et al.^19^ proposed a dual branch GAN based technique for semi-supervised semantic segmentation, as well as a method for using unlabeled images to generate pseudo labels, and then using these for network training to increase performance. Their system achieved 75.6% mIOU (mean intersection over union) on the PASCAL VOC dataset.

Laine et al.^20^ achieved consensus predictions of unknown labels using an ensemble of numerous models trained with various regularizes and augmentation strategies under semi-supervised settings. Applying this method to the SVHN dataset, they were able to minimize the classification error from 18 to 5.12%, using only 500 labelled samples. Sajjadi et al.^21^ proposed a gradient descent-optimized unsupervised loss function that took advantage of randomized data transformation and augmentation to minimize the difference in predictions of multiple passes of a data sample through the network during the training phase, resulting in better generalization during inference. Using only 100 labelled samples, they were able to obtain an error rate of 0.27% when their system was applied to the MNIST dataset.

In lieu of using the final weights of the trained model, Tarvainen et al.^22^ proposed averaging the model weights over different training steps to get improve model robustness. Using this method and only 500 labelled sample, they reduced the error rate to 4.18% when their system was applied to the SVHN dataset. Li et al.^23^ proposed a system that used labeled and unlabeled data, as well as a self-ensembling approach that promoted the network to generate consistent predictions for the same input under different regularizations for skin lesion segmentation. With only 300 labelled samples, they established a new performance benchmark (75.3% mIOU) on the international skin imaging collaboration (ISIC) dataset. Perone et al.^24^ used the Mean Teacher technique, which was first proposed in^22^, to segment MRI images and produced a mIOU of 55.5%, similar to the 53.6% achieved with supervised learning. French et al.^25^ demonstrated that, with the right source of augmentation, consistency regularization is a feasible means of semi-supervised segmentation, as they used a customized CutMix augmentar to produce state-of-the-art results. Their approach was also significantly easier to implement and use than GAN-style training.

Weakly-supervised learning can be divided into three sub-classes: (1) incomplete supervision, in which only a subset of training data is labeled; (2) inexact supervision, in which only coarse-grained labels are assigned; and (3) inaccurate supervision, in which the given labels are not always ground truth. Pinheiro et al.^26^ proposed a weakly supervised framework, in which they generated pixel-level labels of objects in images using only image-level labels provided during training. They trained their CNN to emphasize pixels that played crucial role in classifying the image, and then used different smoothing priors to extend its application to segmentation. Using this strategy, they were able to achieve benchmark performance (weakly supervised segmentation) on the PASCAL VOC dataset. The CAM (channel activation maps) family of algorithms^6,27,28^ determine which portions of an image activate neurons. To improve localization performance, Singh et al.^29^ forced their network to locate more than one discriminative region of an object by blocking out the portion of images at random. To address segmentation problems associated with weak supervision, Wei et al.^30^ proposed an adversarial erasing approach to mine different discriminative object regions. Following this strategy, they were able to achieve 55.7% mIOU on the PASCAL VOC dataset. Other studies have attempted to use attention maps to improve segmentation results^31,32^. Huang et al.^33^ proposed deep seed region growing algorithm to generate segmentation masks. They proposed that a semantic segmentation network be trained first with discriminative areas, followed by gradually increasing pixel-level supervision through seeded region growth^34^. On the PASCAL VOC dataset, their system achieved a 66% mIOU using this method. Alternatively, identifying segmentation regions using box annotations that contain object has also been studied^35,36^. A summary of state-of-the-art algorithms for easy understanding can be found in Table 1.

**Table 1:** Summary of state-of-the-art algorithms in domain of weakly and semi superivsed framworks for image segmentation.

Framework	Algorithm	Key points
Semi-supervised	Fully Convolutional Discriminator^13^	The authors utilized a discriminator network^14^ to train a CNN for semantic segmentation task using both labelled and unlabeled images The network was optimized by coupling adversarial and standard cross entropy loss
	Dual-branch GAN^19^	The authors generated pseudo labels from unlabeled images, which were then used to train the network Final segmentation masks were refined by MLMT (multi-label mean teacher)^23^ sub-network to improve performance
	Self-Ensembling Model^23^	The algorithm enabled the network-in-training to provide consistent predictions for the same input under different regularizations the network was optimized via weighted combination of supervised loss (labeled data) and a regularization loss (labeled + unlabeled data)
Weakly-supervised	Overfeat + Pixel-wise Segmentation^26^	Firstly, they generated pixel-level labels of objects in images using only image-level labels provided during training Then used different smoothing priors on pixels, that played crucial role in image classification, to generate segmentation masks
	Hide & Seek^29^	Trained the network by forcing it to locate more than one discriminative region of an object by blocking out the portions of an image at random
	Adversarial Erasing^30^	Used adversarial erasing approach for progressively mining the discriminative object regions during classification Then used these mined regions to generate complete dense objects
	Region Growing^33^	In contrast to conventional segmentation algorithms that use static labels, the authors here used seeded region growth algorithm^34^ to generate new labels during each training cycle
	BoxSup^35^	For network training, bounding box annotations were utilized to produce candidate masks using unsupervised region proposal methods^37,38^ These candidate masks improve with each iteration, providing more and more valuable information for CNN training

Semi-supervised learning that uses a combination of labeled and unlabeled samples can enhance a model's performance. The generation of a segmentation label for a plant, even for a single image, is a time-consuming job (as illustrated in Fig. 1). When very little data is available for training, weakly supervised algorithms are available, though these are difficult to implement, train, validate, and quantify. Moreover, semi- and weakly-supervised algorithms have complex model structures and frequently use pre-trained models to increase performance. In the agricultural domain, however, the use of pretrained models is difficult as pretraining is normally done with a dataset from a completely different domain. Only a few models have been trained with agricultural data.

Different from these prior methods, we created MGRL that pertain to the same domain and utilized them to guide the CNN training. The trained model was able to create PLFLs quickly and accurately for a given data sample. Furthermore, because MGRLs are easier and faster to generate than PLFLs, they are more swiftly organizable into the sort of data that is needed for learning.

## Materials and methods

Gathering the data to train a CNN for precision agriculture and smart farming is more difficult than gathering the data for other tasks. The process is complicated by each plant's intricate geometry and the overlapping nature of leaves, branches, and fruits. Furthermore, weeds, land, sunlight, shadows, and wind affect crops, and can make it difficult to distinguish crops from surrounding items during labeling^6^. Many crops have a shape similar to that of weeds and can only be distinguished by an expert^4^. These problems increase the cost of building a dataset and delay training based on new data. Some studies have successfully attempted to overcome this hurdle by utilizing synthetic data generation^39^. But synthetic images are inherently different from actual images^40^. As a result, when trained using synthetic images and deployed in a real-world setting, DNNs that are sensitive to even minute changes in the input may see a performance reduction^41^. Therefore, we propose a labeling strategy depicted in Fig. 2 to overcome this fundamental difficulty of preparing segmentation labels. We validate our model on several datasets having a variety of backgrounds and environmental conditions, as summarized in Table 2.

**Table 2:** Dataset properties.

Dataset	Type	Environment	Background
Circle dataset	Synthetic	–	Uniform
Bean-field dataset	Authentic	Outdoor	Simple
Paprika-disease dataset	Authentic	Outdoor	Complex
CVPPP^42^	Authentic	Indoor	Simple
CWFID^43^	Authentic	Outdoor	Simple

As discussed further in ″Manually Generated Rough Labels (MGRL) and Channel Difference Threshold Labels (CDTL)″ Sections, the final PLFL requires a pair of pseudo labels, one generated manually (i.e., MGRL) and the other generated automatically by thresholding the RGB channel difference through CDTL. The segmentation model can then be trained with these coarse label pairs (CLPs), to generate pixel-level fine labels (PLFL).

### Datasets

We created three new datasets to test the proposed technique: Circle, Bean-Field, and Paprika-Disease datasets. Additionally, we also applied the technique to the publicly available CVPPP^42^ and CWFID^43^ datasets. All images were downsized to 512 × 512 without regard for aspect ratio.

#### Circle dataset

To test the proposed method for producing fine labels, basic circular objects with gaussian noise were randomly put on a noisy pallet, as shown in Fig. 10. The Circle Dataset had 500 data samples in total. In this case, MGRL had a somewhat wider diameter than their corresponding true circular objects.

#### Bean-field dataset

**Figure 3:**
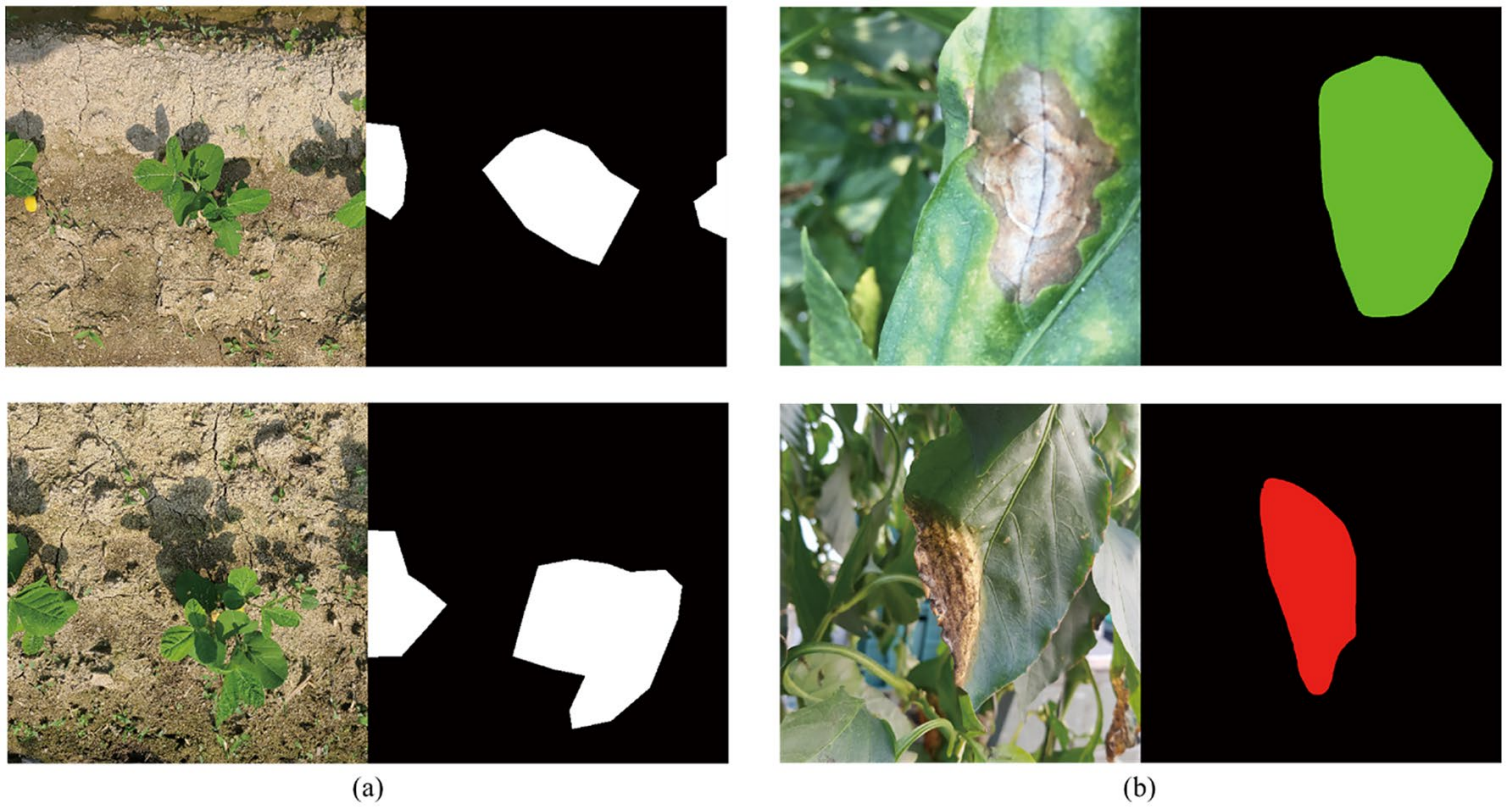
Input data sample and corresponding manually generated rough labels (MGRLs), (**a**) Bean-Field Dataset, (**b**) Paprika-Disease Dataset.

The bean field dataset employs images gathered on a private soybean farm in Gimje, Jeollabuk-do, South Korea. It was filmed from a top-down perspective and consists of a total of 252 photographs. A sample image along with its MGRL is shown in Fig. 3a. The field was photographed three times per week for 5 weeks, at the beginning and middle stages of plant development. The photographs were taken in a variety of environmental conditions, including shadow, lightning, rain, and clouds.

#### Paprika-disease dataset

Images of paprika leaves infected with three different diseases—blossom-end rot, spotting disease, and gray mold—are included in the dataset. The dataset was collected at the JBARES Paprika Test Site, which is run by the South-Korean Research Development Authority (RDA) and consists of 90 images in total. Figure 3b shows a few sample shots from the dataset, which includes side views of paprika leaves.

### Pixel level fine labels (PLFL)

A fine label is a pixel-precise label that is used in general artificial neural network training. Using tools like LabelMe^44^, semantic segmentation labels can be created for multiple categories with pixel-level precision. Each label is typically created in the form of a polygon, which consists of vertices and edges. The more complex and wider the boundaries of the object to be annotated, the longer its creation takes. The semantic segmentation label of a single instance from the Bean-Field dataset is shown in Fig. 1c.

### Manually generated rough labels (MGRL)

The convex that contains or envelopes the approximate region of an object in an image is referred to as a manually generated rough label. Samples of such convex regions (rough label) is shown in Figs. 1b and 3. To speed up production, the convex, while it should encompass the entire object, does not have to closely follow the boundary of the object it contains. It can be applied not only to crop images, but also to diseases that manifest on leaves, stems, and fruits. Figure 3 depicts a few manually generated rough label (MGRL) samples corresponding to the input image.

### Channel difference threshold labels (CDTL)

**Figure 4:**
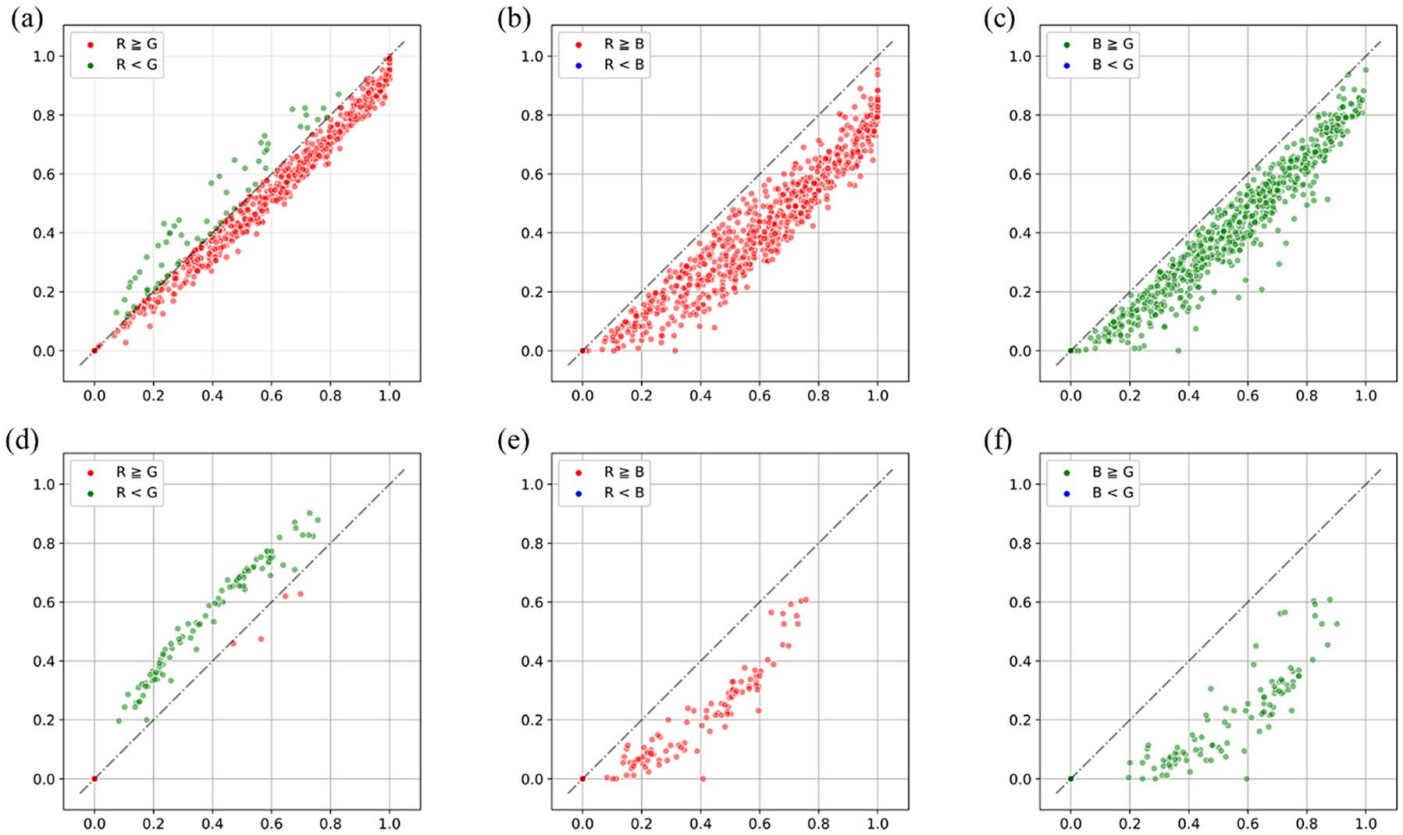
Distribution between channels of pixels in crop images. (**a)**, (**b)** and (**c**) show the object; (**d)**, (**e)** and (**f**) show the background.

Channel differential threshold labels (CDTL) for a given data sample can be generated automatically provided that the corresponding MGRL is available. For a representative data sample from the Bean-Field dataset, Fig. 4a depicts a scatter plot between the normalized pixel values of two channels i.e., between R and G channel (R-G). Here R, G, and B correspond to the red, green and blue channel of an RGB image. In Fig. 4a the x-axis displays the intensity of the R-channel, and the y-axis displays the intensity of G-channel. The black dotted line in the scatter plots is drawn where x = y (i.e., where pixels have same value in both channels). The other scatter plots in Fig. 4 similarly plot the pixel values of the remaining two RGB-channel pairs (R-B and B-G). Figure 4a,b,c plot the intensities of pixels constituting the foreground object (bean plant). Figure 4d,e and f plot the intensities of pixels that make up the entire background (soil, weeds, etc.).

As can be seen from Fig. 4b,c,e and f, the distribution of R-B and G-B pair pixel values makes it difficult to distinguish between foreground and background objects using channel values. However, in the case of the R-G pair (Fig. 4a,d), foregrounds and background pixels can be roughly distinguished. Because the foreground object is a plant, it has higher pixel values in its green channel than in its red channel.

In sum, CDTLs are generated by classifying an object region based on color attributes. The red and green channels of the input image are divided by the maximum value of each channel and each pixel position is converted into a relative intensity between 0 and 1. For example, in the bean leaf region, the intensity of the G-channel is stronger than that of the R-channel. In the background region, the R-channel has a stronger or a similar intensity to that of G. Therefore, it is possible to identify an alpha value that satisfies the following Eq. 1.

1 $$ \begin{array}{*{20}c} {\begin{array}{*{20}c} {Object \leftarrow G^{\prime }_{i,j} - R^{\prime }_{i,j} \ge \alpha } \\ {Background \leftarrow G^{\prime }_{i,j} - R^{\prime }_{i,j} < \alpha } \\ \end{array} } & {\forall \left( {i,j} \right) \in \left( {m,n} \right)} \\ \end{array} $$

in which (*i, j*) represents the location of the pixel on a *m* x *n* resolution image. The values of *G′*_*i,j*_ and *R′*_*i,j*_ can be obtained using;

2 $$ G\prime_{i,j} { } = \frac{{G_{i,j} }}{\max \left( G \right)} $$

3 $$ R\prime_{i,j} { } = \frac{{R_{i,j} }}{\max \left( R \right)} $$

**Figure 5:**
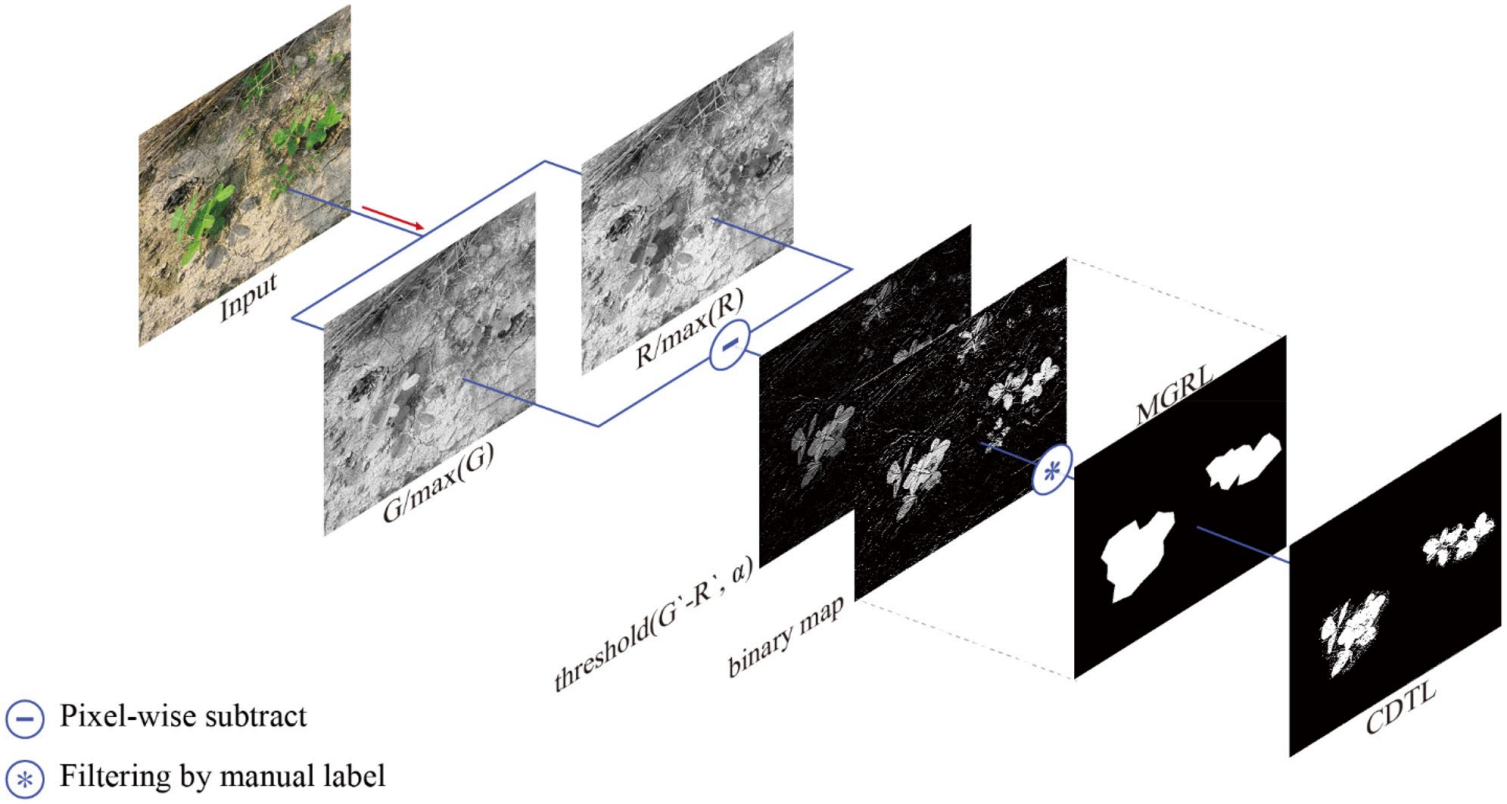
Pipeline for generating channel difference threshold label (CDTL).

The value of α, obtained from Eq. 1, can be used as a threshold to generate a binary map that highlights the foreground and background regions of the given data sample. Finally, the CDTL can be generated on the basis of the binary map by keeping only the values within the MGRL while zeroing out the others. Mathematically, this process can be represented as follows.

4 $$ \begin{array}{*{20}c} {\begin{array}{*{20}c} {P_{i,j} = Thrshold\left( {G^{\prime }_{i,j} - R^{\prime }_{i,j} ,\alpha } \right)} \\ {P\prime_{i,j} = P_{i,j} \& C_{i,j} , \forall \left( {i,j} \right)} \\ \end{array} } & {\forall \left( {i,j} \right) \in \left( {m,n} \right)} \\ \end{array} $$

in which *P*_*ij*_ stands for the binary threshold map, *C*_*ij*_ stands for MGRL, and & stands for pixel-wise “*and*” operation. Figure 5 presents the CDTL generation process in its entirety.

### Generalized channel difference thresholding (GCDT)

**Figure 6:**
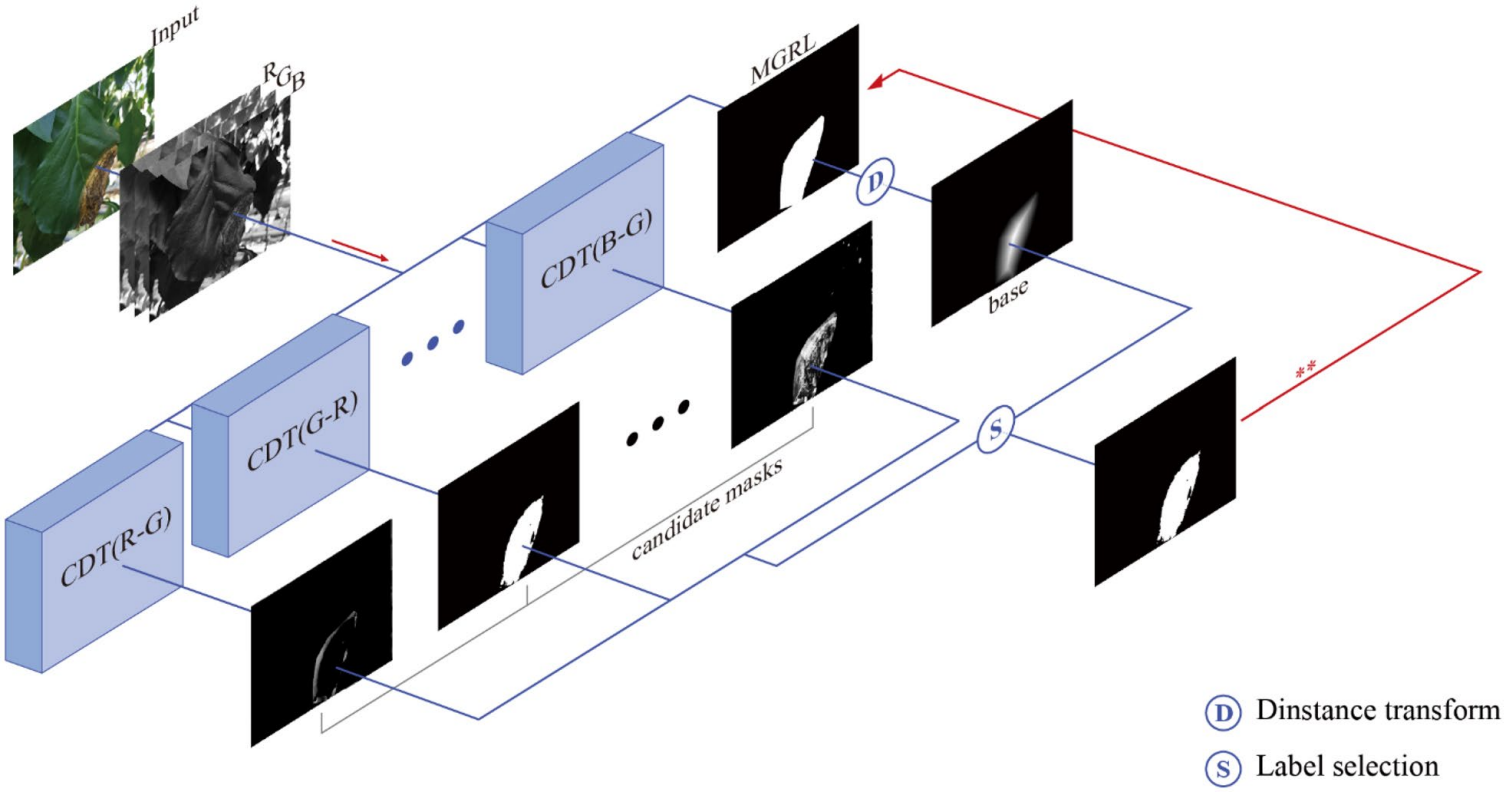
The overall pipeline of the generalized channel difference thresholding (GCDT) algorithm.

Channel difference threshold labelling was designed specifically to generate CDTL masks for the Bean-Field dataset. When this method was generalized and applied to other datasets in which the pixel intensities of all the channels were randomly spread out over the spectrum, for example, in case of disease recognition in Paprika-Disease dataset, it had difficulty distinguishing between foreground and background pixels. In this section we propose a method of generalizing the CDTL algorithm such that it can be readily applied to various datasets possessing multiple classes. In contrast to CDTL, generalized channel difference thresholding (GCDT) generated binary maps of all possible RGB-channel pairs (R-G, B-G, and G-R). The overall pipeline of the GCDT method is shown in Fig. 6.

GCDT generates threshold labels that correspond to each channel pair through iterative operations as shown in Fig. 6. Distance transform^45^ is applied to the annotation corresponding to a specific class in the manual label (MGRL). The optimal threshold label is selected by comparing the similarity of the created distance mask (base) with all the candidate threshold labels. To obtain a multi-class threshold label, this method is performed for each class in the MGRL. To identify the optimum threshold label for a certain class, we employ IOU as a similarity measuring metric. Before measuring the IOU, a threshold of 0.7 is applied to the distance transform.

## Data configuration and validation strategy

**Figure 7:**
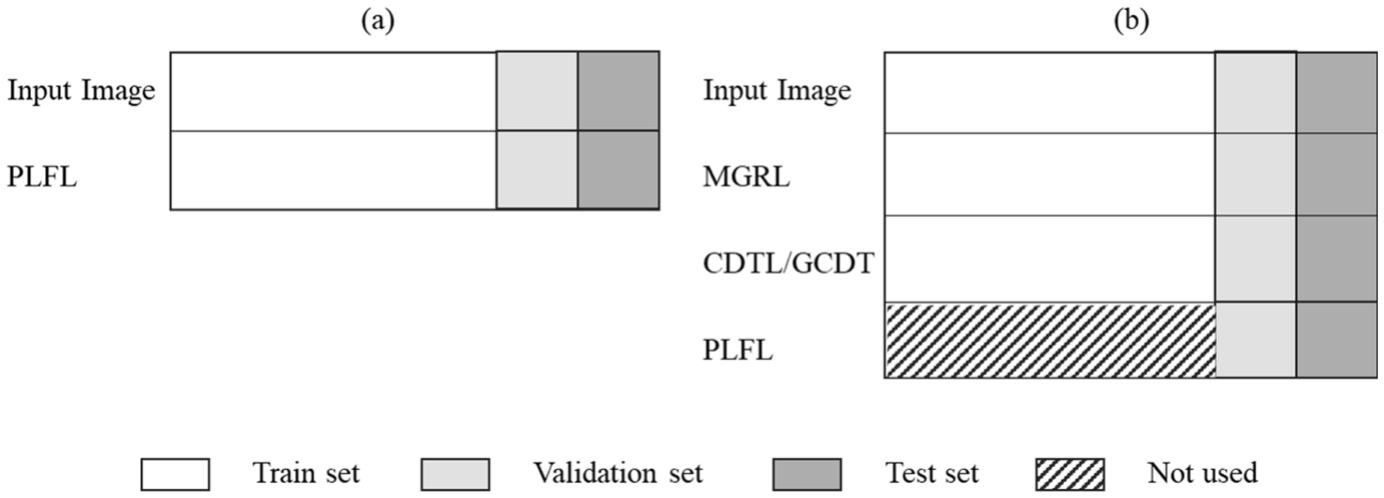
Distribution of data for conventional training and the proposed training method. (**a**) Data distribution in general neural network training. (**b**) Data distribution in the proposed method.

In traditional NN training, the robustness of a model against unseen data is validated by partitioning the data set into train, validation, and test sets (Fig. 7a). We used validation data to measure the generalization of the trained model and tweak parameters, while a test set was used to evaluate the performance of trained model.

**Figure 8:**
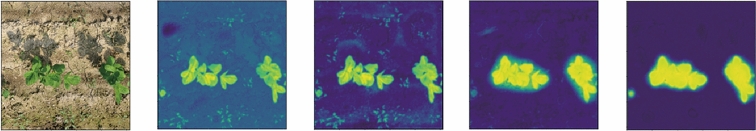
The output of the model at different epochs when training with MGRL. From the left to right, the boundary within the area is captured, but eventually this becomes similar to the manual label.

Unlike conventional neural network training in which the goal is to make predicted label’s hew as closely as possible to target labels, our technique aimed to generate fine labels with coarse labels as targets. If the training duration was too long or short, the generated labels would be biased toward over- or under-segmentation. Figure 8 reflects how, if the training period was too short, the labels created would not cover the entire object, however if the training time was too long, the generated labels would resemble MGRLs. Selection of the proper training end point was therefore critical. In the proposed method, the fine labels of some images were generated and utilized as a validation set to identify this optimal training end point. Unlike traditional neural network learning, in which the validation and training data are separated, we included the validation data in the training data. At each iteration, the IOU for this validation set was calculated to determine the best training end point. Fine labels for the entirety of the image datasets were generated during these experiments to confirm the validity of this strategy.

## Segmentation model and pre-validation

### Segmentation model

U-Net^46^ was adopted as the baseline model in our experiments. U-net is a fully convolutional encoder–decoder network, characterized by a simple ‘*U*’ shaped structure, fast training, and pixel level dense predictions with an output close to the resolution of the input image. We modified the concatenation paths of a U-Net to control the flow of information between its encoder and decoder. More precisely, instead of simply concatenating the encoder (*E*_*ij*_) and decoder (*D*_*ij*_) features, we first reweighted the incoming features using a constant *γ* and then performed element-wise addition. Mathematically this process can be written as,

5 $$ \left( {1 - \gamma } \right)E_{i,j} \oplus \gamma .D_{i,j} $$

in which ⊕ denotes element-wise addition between. The value of *γ* was set at 0.2. In this study U-net was set as the baseline, but other fully convolutional neural networks (FCNs) can also be used.

### Hypothesis and pre-validation

Humans generate segmentation labels inconsistently and are therefore not perfect at performing this task^47–49^. Unlike an ideal fine label, therefore, the fine labels produced by this process may contain many false positives or false negatives. However, these inaccuracies can be considerably suppressed when labels are generated by FCN.

We assumed that a training model using manual label would be possible due to the generalization ability of the NN and the low-density separation of data^50,51^. The object's border was not precisely defined in the manual label, but the density of the data distribution that appropriately defined each class of object was greater than the density of the boundary region. This means that the density of the decision boundary in the feature space was minimal, which allowed the network to learn along the exact object boundary when training. Figure 8 shows the output at different epochs while the aforementioned segmentation model was trained using CLPs (i.e., MGRL + CDTL). The model was trained for a total of 25 epochs and input images were normalized between 0 and 1. The mean squared error loss function and Adam optimizer were used for training the network. While CLPs were used as targets during the training, leaf boundary was accurately predicted during training process. This experiment confirmed that even if a model trained using CLPs, reduced the number of false positives or false negatives, and that an object’s boundary information could be inferred from input data. However, as the training proceeds in a different direction than intended due to incorrect annotation (i.e., MGRL), the final results get closer to the MGRL, like the output after 25th epoch shown in Fig. 8.

Therefore, we devised a threshold label that interacts with the manual label to assist with predicting the precise boundary of an object. The threshold label is generated using the GCDT. Finally, with manual label (MGRL) and threshold label (CDTL), the model is trained using the objective function, shown in Eq. 6.

6 $$ L_{all} = L(O_{i,j} ,C_{i,j} ) + L\left( {O_{i,j} ,P_{i,j} } \right) \forall \left( {i,j} \right)) \in \left( {m,n} \right) $$

here *O*, *C*, and *P* denote the output of the artificial neural network, manual, and threshold label respectively. *L* is determined as the mean squared error for a single class and as cross entropy for multiple classes, with *L*_*all*_ being the overall loss function.

**Figure 9:**
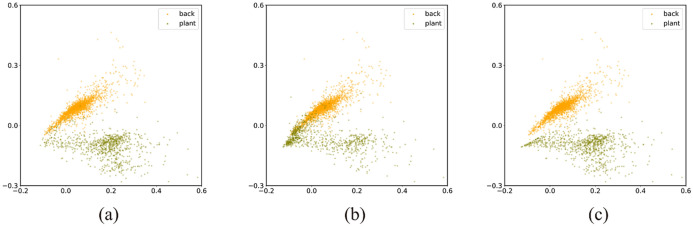
Distribution of data in two-dimensional feature space according to different labelling methods. (**a**) PLFL, (**b**) MGRL, (**c**) CDTL. Here, ‘back’ stands for background (soil, weeds, etc.) and ‘plant’ stands for foreground (bean plant).

Figure 9 depicts the distribution of data from the Bean-Field dataset in two-dimensional feature space according to the labeling approach utilized, i.e., fine, manual, or threshold labeling. Figure 9a shows the distribution of data for fine labels (PLFLs). In this case, the data is clearly demarcated, and a decision boundary can easily be formed. Due to the properties of the incorrect labels, manual labeling (MGRL) increased the relative density of data at the optimum decision boundary, as illustrated in Fig. 9b. Ultimately, due to the large number of clearly differentiating traits, a decision boundary similar to that of a fine label can be constructed. The density of the decision boundary was lowered in the case of the threshold label (CDTL), as shown in Fig. 9c, since the change in parameters caused by the inaccurate characteristics of the manual label was corrected by the properties corresponding to the correct label. In other words, the inclusion of a manual threshold label allows for the generation of a more certain decision boundary.

**Figure 10:**
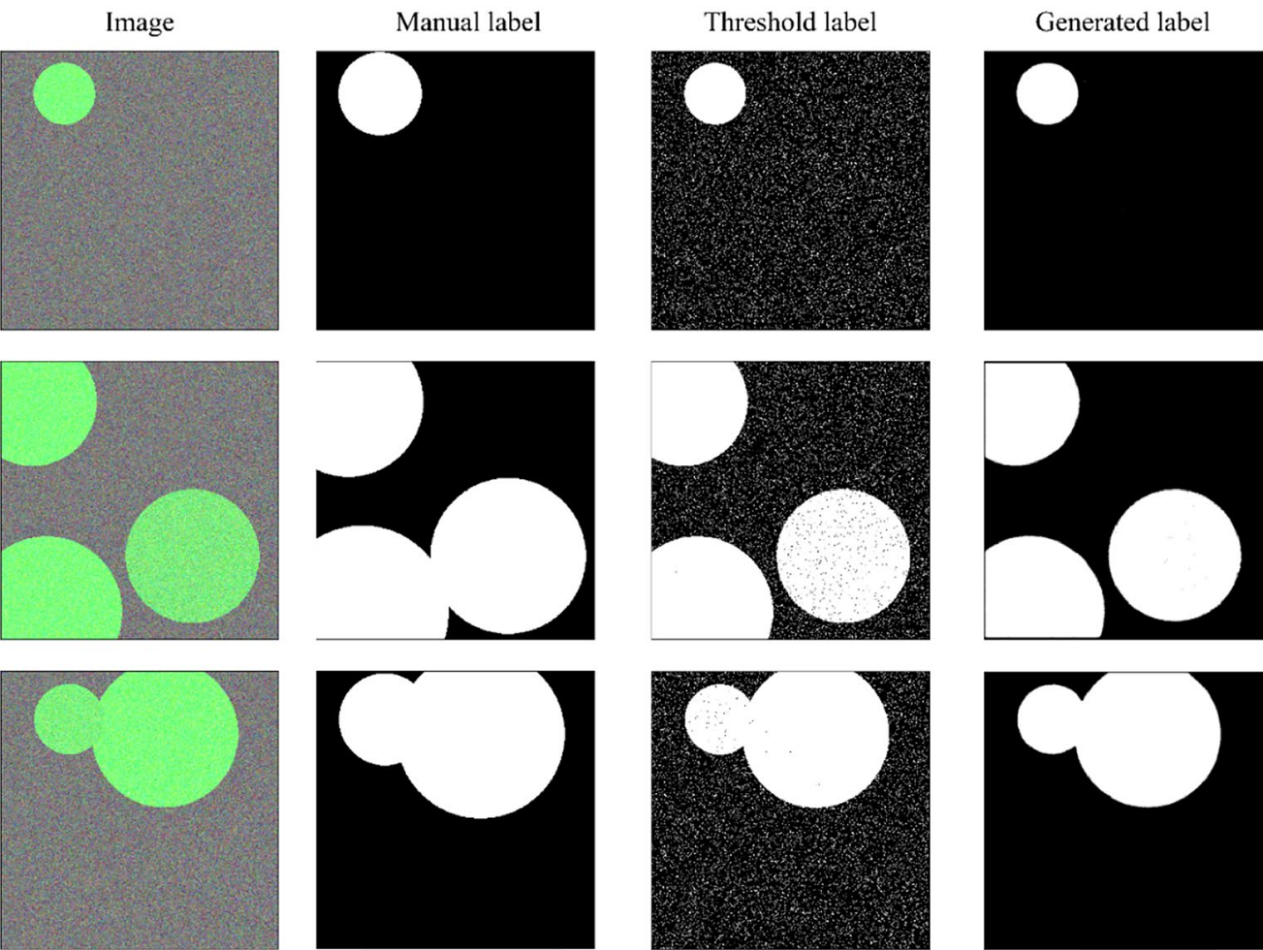
Training results on the Circle dataset. Annotations inside and outside the object were corrected through the interaction of MGRL and CGTL.

A circle dataset was constructed, and a preliminary experiment was conducted to examine the combined effect of CLPs on a CNN’s training. Training was carried out for 5 epochs considering the characteristics of the dataset. Figure 10 presents the training results when both labels were used. While the diameter of the MGRLs was somewhat greater than the diameter of the actual circular object, the item's border line was precisely predicted, and it closely followed the original object's boundary.

## Experiments and results

### Evaluation metrics

For performance measurement of our proposed approach, we utilize following widely used evaluation metrics in segmentation literature.

#### Intersection over union (IOU)

IOU also known as the Jaccard Index, is one of the most used straightforward and effective metrics in semantic segmentation. IOU quantify the percent overlap between the ground truth mask and network’s prediction. IOU measures the number of foreground pixels (positive) common between the target and prediction segments divided by the total number of pixels present across both segments. In our experiments Eq. 7 is used to measure IOU of class *c*.

7 $$ mIOU_{i} = \frac{1}{C}\mathop \sum \limits_{c} IOU_{c} = \frac{{TP_{c} }}{{FP_{c} + FN_{c} + TP_{c} }} $$

In case of binary segmentation, TP represents the number of foreground (positive) pixels correctly predicted as foreground, FP represents the background (negative) pixels wrongly predicted as foreground and FN represents background pixels wrongly predicted as foreground.

#### Precision (P)

Precision shows us what proportion of all detected foreground pixels were actually true positives (TP). Equation 8 is used to measure precision.

8 $$ Precision = \frac{{TP_{c} }}{{FP_{c} + TP_{c} }} $$

#### Recall (R)

Recall indicates that, out of all the foreground pixels present in an image how many of them were correctly predicted. It is computed via following Eq. 9.

9 $$ Recall = \frac{{TP_{c} }}{{FN_{c} + TP_{c} }} $$

#### F1-score

It can be defined as the harmonic mean of precision and recall, as given in Eq. 10. Since the F1-score considers both precision and recall, it accounts for both FPs and FNs.

10 $$ F1score = \frac{{2TP_{c} }}{{2TP_{c} + FN_{c} + FP_{c} }} $$

#### Matthews correlation coefficient (MCC)

Just like F1-score, MCC is a single-valued metric that sums up the performance of the network. But unlike F1-scor it does not ignore the effect of TNs (number of background pixels correctly predicted as background) on networks performance. Which means that the value of MCC is high only when the network performs well in both foreground and background cases. It can be calculate using following Eq. 11.

11 $$ MCC = \frac{{TP_{c} \times TN_{c} - FP_{c} \times FN_{c} }}{{\sqrt {\left( {TN_{c} + FN_{c} } \right)\left( {FP_{c} + TP_{c} } \right)\left( {TN_{c} + FP_{c} } \right)\left( {TP_{c} + FN_{c} } \right)} }} $$

### Binary class label generation

The model was first trained on the Bean-Field dataset for 40 epochs with a batch size of 12, a learning rate of 0.005, and a prediction threshold of 0.7. Table 3 summarizes our framework's performance in comparison to other state-of-the-art semi-supervised segmentation algorithms such as AdvSemiSeg^13^ and BoxsUp^35^, which can be trained and validated in the same way. Our method produced an overall PLFLs having mIOU of around 92%, compared to 82% and 85% for BoxsUP and AdvSemiSeg, respectively.

**Table 3:** Comparison with state-of-the-art semi-supervised segmentation algorithms.

Framework	Architecture	mIOU (%)	Precision (%)	Recall (%)	F1-Score (%)
Boxsup	U-Net	82.16	87.47	93.50	90.38
Boxsup	DeepLab v3	82.54	84.66	**96.09**	90.02
AdvSemiSeg	DeepLab v2	85.48	89.81	93.29	91.52
Proposed	U-Net	**92.43**	**96.84**	95.31	**96.07**

The learning strategy and verification of all results were carried out in the same way as in Fig. 7.

Best results are shown in [bold].

**Figure 11:**
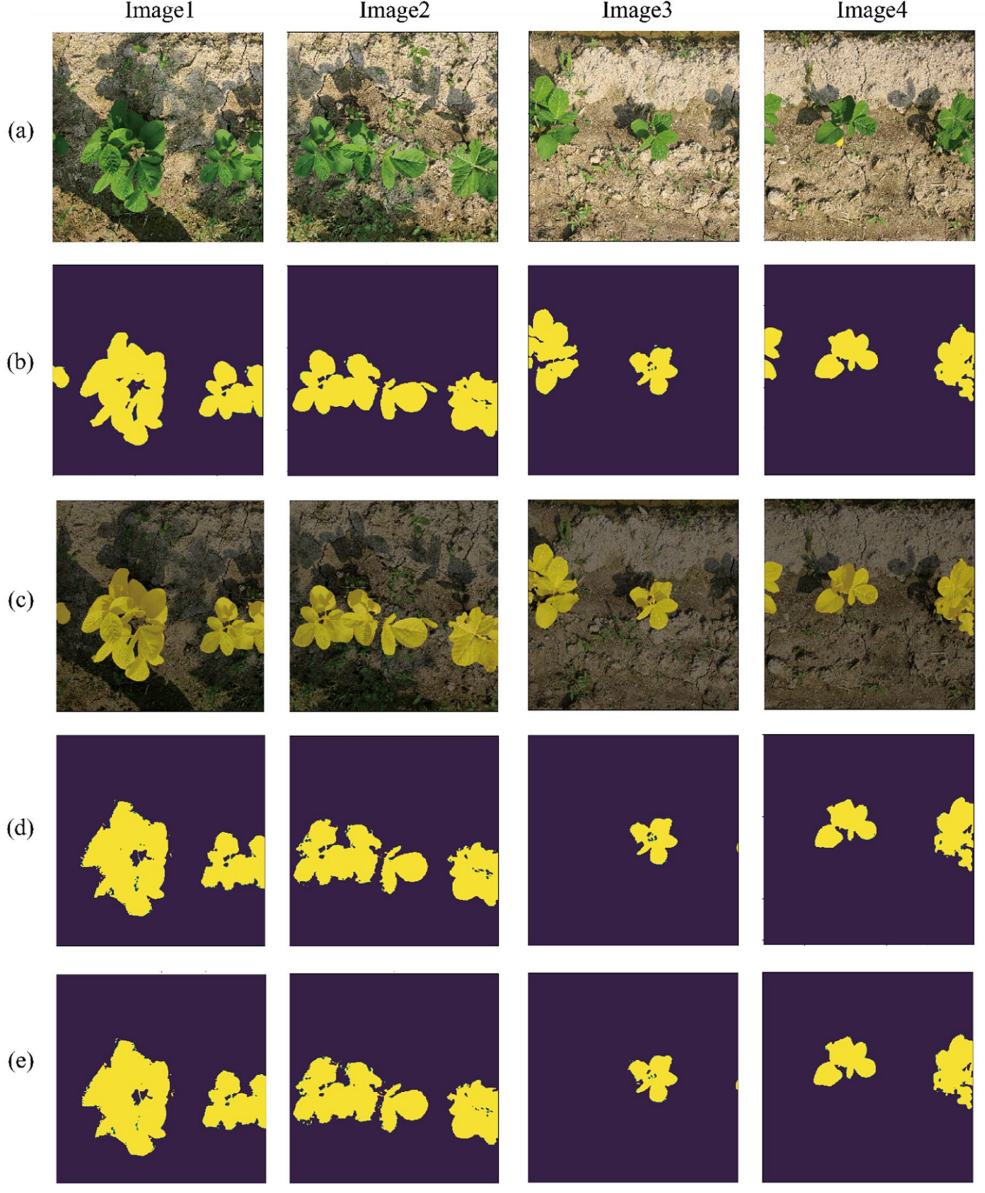
Qualitative Comparison of output by the proposed method and filter on the Bean-Field dataset. (**a**) the input image, (**b**) the output of the proposed method, (**c**) overlay of the output on the input image (**d**) output when average filter is applied and (**e**) output when a median filter is placed on a CDTL.

**Figure 12:**
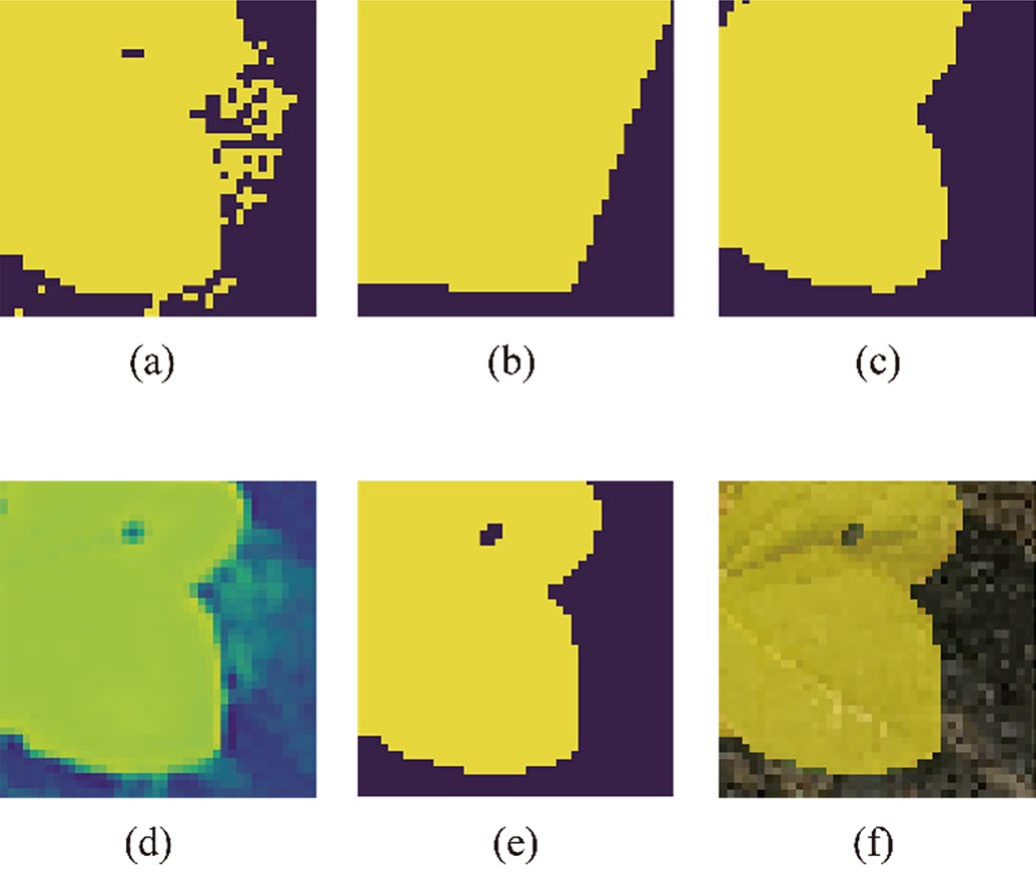
Local output results for the Bean-Field dataset. (**a**) threshold label, (**b**) manual label, (**c**) fine label, (**d**) output of the model, (**e**) output on which a threshold is applied, (**f**) overlay on the input image. In the output image of the trained model, the noise seen in the threshold label is well removed and the holes on the leaf are accurately indicated.

The algorithm's performance was then compared to the case when the noise from the CDTLs were directly removed using a low-pass or median filter. Table 4 summarizes these findings. Employing filters on the CDT label to eliminate noise caused a minor change in mIOU. However, as reflected in Fig. 11, qualitative findings vary dramatically depending on the application of average/median filter. Figure 11 provides samples of the results (on Bean-Field dataset) achieved in each column. Figure 11b depicts the algorithm's predictions without any filtering used. Figure 11d,e illustrate the results of using average and median filtering on CDTLs, respectively. The application of noise removal filters resulted in some objects not being detected in the final predictions, as seen in column’s three and four of Fig. 11. The proposed method inherently detected the object boundaries, filled the holes in the predictions, and suppressed noise at the boundary. Figure 12 shows the local results of these effects.

**Table 4:** Comparison of the proposed method with and without the application of noise removal filter on the CDTLs.

Architecture	Filter	mIOU (%)	Precision (%)	Recall (%)	F1-Score (%)
U-Net	–	**92.43**	96.84	95.31	**96.07**
U-Net	Low Pass	91.58	95.15	**96.05**	95.60
U-Net	Median	91.73	**97.69**	93.74	95.68

Best results are shown in [bold].

The time required for label production, as well as to achieve accurate labeling, is critical. Table 5 presents how long it took to manually (by hand) generate PLFLs for the Bean-Field dataset as well as how long it took to semi-automatically generate labels using the proposed method. When two TITAN-RTX units were used, the total training time, including verification and threshold label generation, was approximately one hour, with label generation taking around 15 min. For some samples manual label generation time was estimated to be around 2 min for MGRL and about 18 min for PLFL, on average. Even after the time required for training is factored in, the generation time for the Bean-Field fine label achieved by the proposed method was at least 60 h less than that required for hand labeling. The factors that account for this difference were labeling experience and the shape of the objects present in the image. Furthermore, as all input data was labeled at the pixel level during the training phase, annotations with more accurate boundaries than those achievable using semi- and weakly-supervised system were generated.

**Table 5:** Comparison of time required for labeling by manual means and the proposed method for a few samples.

Type	Vertices/sample	Labelling	Training	Total
		(min/sample)		(min/sample)
Hand-made	387.2	18	–	18
Proposed	33.9	2	0.24	2.24

### Ablation studies

We performed several ablation experiments to assess the performance of each component of our proposed algorithms, and the results are presented in Table 6. Table 6 shows that when MGRLs and CDTLs were jointly utilized for network training, results were improved by approximately 3% compared to the case when these were used separately. Furthermore, compared to standard U-Net style concatenation, the results achieved using our modified feature fusion mechanism (described by Eq. 3) were improved by 2%.

**Table 6:** The performance of the model using various combinations. The results are best when CDTL, MGRL, and attention connections are used in combination.

MGRL	CDTL	Attention connection	mIOU (%)	F1-Score (%)	MCC (%)
✓			87.6	93.4	75.8
✓		✓	88.9	94.1	76.71
	✓		90.6	95.0	89.3
	✓	✓	90.8	95.2	90.8
✓	✓		90.7	95.1	90.8
✓	✓	✓	**92.4**	**96.1**	**95.4**

Best results are shown in [bold].

As MCC metric also accounts for TNs, using only MGRL for training results in higher background errors as the labels coarsely envelop the objects. In contrast, while using only CDTL for training the MCC improves by about 13% showing that network trained with CDTL labels made fewer background mistakes. The MCC does not appear to improve much when both labels are used together, but the addition of attention connection improves the network's performance by nearly 21% over baseline.

### Experiments with public dataset

**Figure 13:**
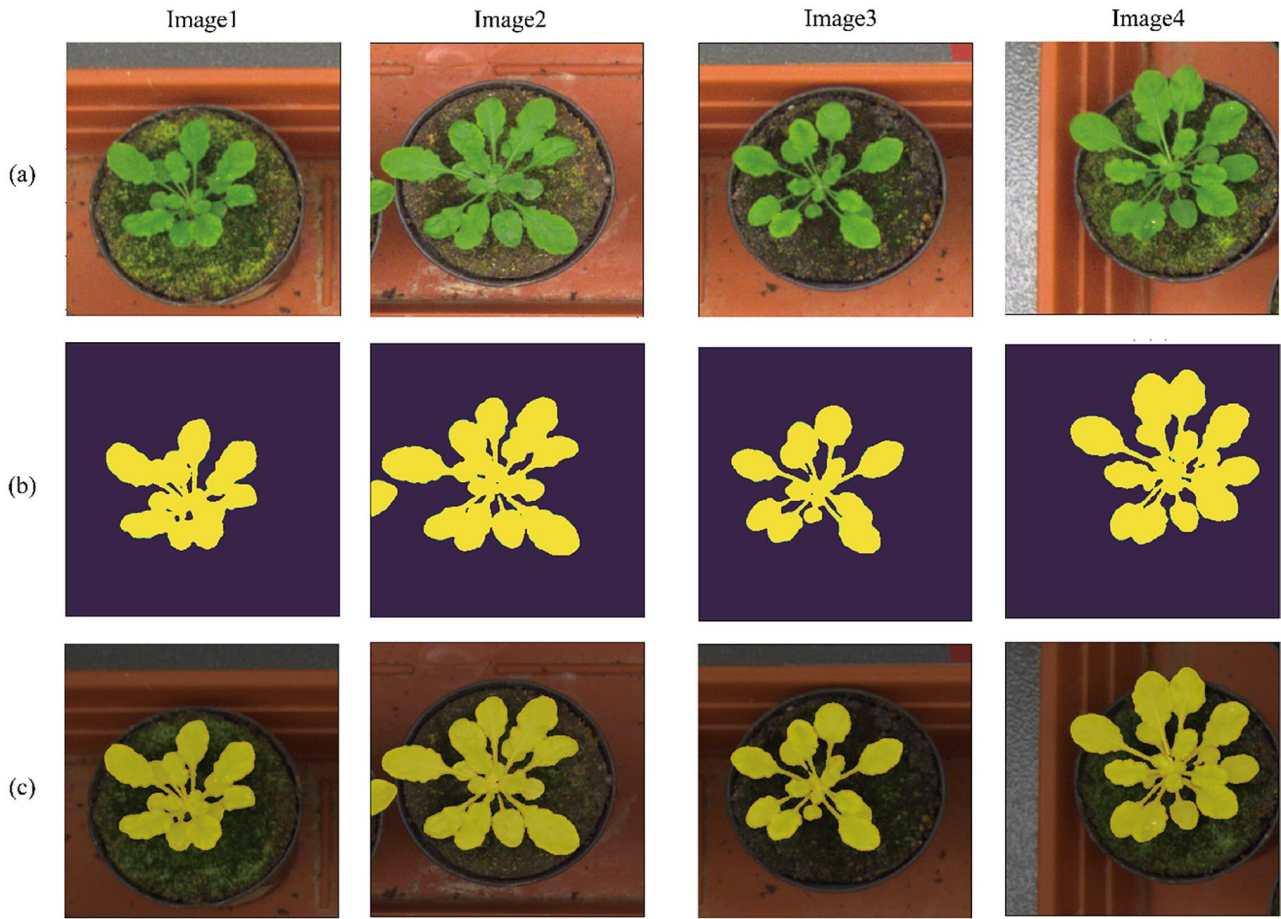
Qualitative results on CVPPP dataset. (**a**) input image, (**b**) output of the proposed method, and (**c**) the overlay image.

**Figure 14:**
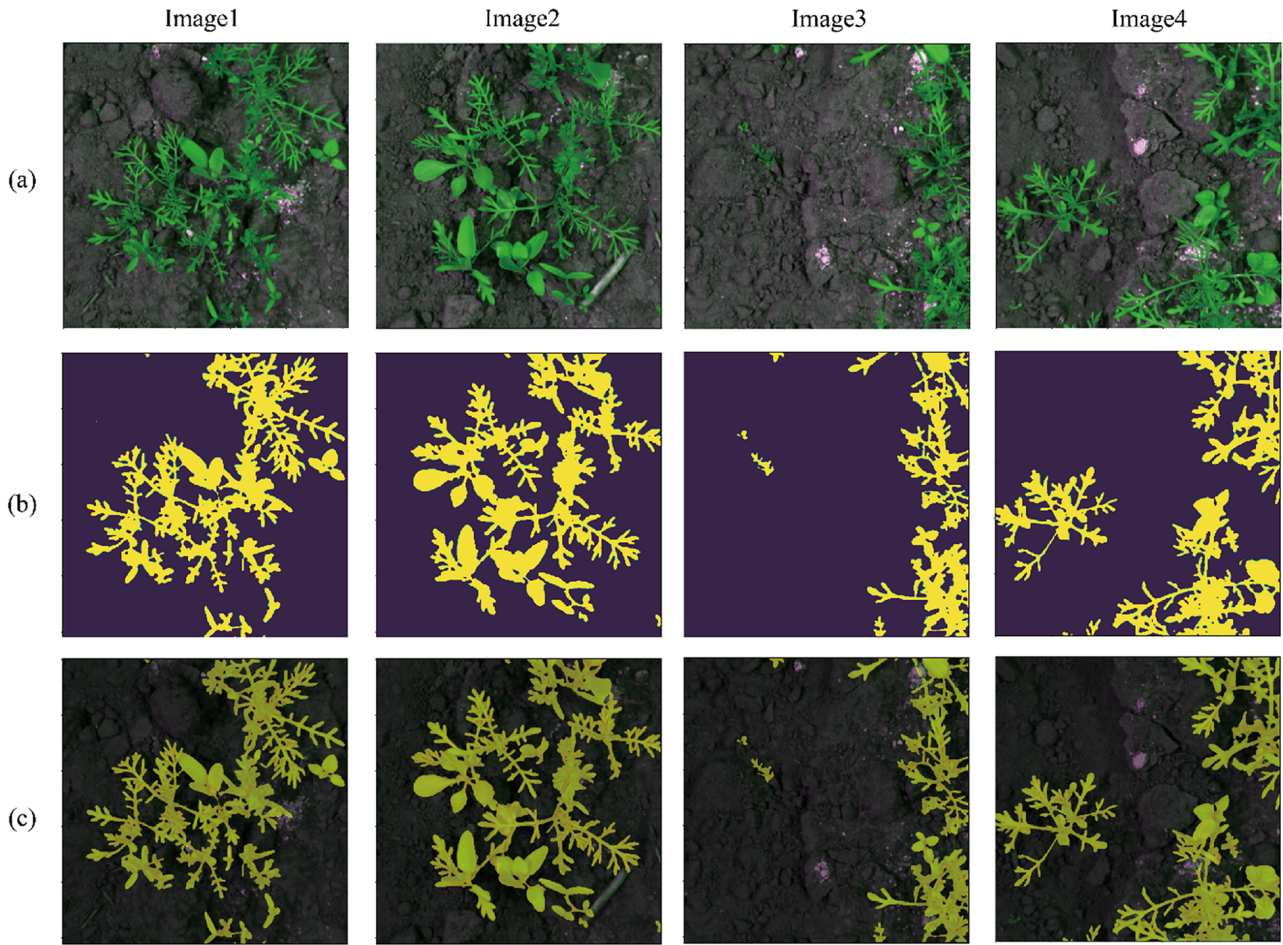
Qualitative results on CWFID dataset. (**a**) input image, (**b**) output of the proposed method, and (**c**) overlay image.

To determine whether the proposed method could be applied to other datasets, we tested it using CVPPP and CWFID. The training was carried out over 50 epochs. Other parameters were identical to those for the Bean-Field dataset. The algorithm's quantitative and qualitative performance with the CVPPP and CWFID datasets is shown in Table 7, Figs. 13 and 14. Our system was able obtained 90% (with CVPPP) and 86% (with CWFID) mIOU. Furthermore, as illustrated in Figs. 13 and 14, precise boundaries were produced for both simple and complicated crops.

**Table 7:** Segmentation labeling result; applying the proposed algorithm to the CWFID and CVPPP datasets.

Dataset	mIOU (%)	Precision (%)	Recall (%)	F1-Score (%)
CWFID	90.67	93.01	97.36	95.14
CVPPP	86.86	99.58	87.18	92.97

### Label suitability assessment

The most appropriate criterion for label suitability assessment would be to measure the similarity between predicted segmentation masks while using PLFLs generated by our approach and PLFLs supplied by human annotators as targets for training the CNN. To see if the PLF labels generated by our system could be used as training targets for CNN, various benchmark CNNs were trained twice. In the first training, we used PLF labels generated by our algorithm as targets. In the second training, we used manually generated PLF labels as CNN training targets.

We used the Bean-Field dataset in these experiments. The ratio of train: validation: test sets was 8:1:1 and mean IOU was used as evaluation metric. The CNNs were trained for 60 epochs under the same conditions, with a learning rate of 0.005. The results are presented in Table 8, in which ‘Manual-PLF’ denotes the use of manually generated PLFLs as targets and ‘Auto-PLF’ denotes the use of algorithm generated PLFLs as targets.

**Table 8:** Label suitability assessment through multiple segmentation networks.

Model	mIOU (%)		
	Manual PLFL	Auto PLFL	Similarity rate
FCN_ResNet50^52^	88.18	87.58	99.3
FCN_ResnNet101	87.18	86.94	99.7
DeepLabV3_ResNet50^53^	87.92	86.66	98.5
DeepLabV3_ResNet101	87.76	87.62	99.8
DeepLabV3_MobileNetV3	79.25	77.54	97.8
LR-ASPP_MobileNetV3^54^	87.20	88.28	**99.99**
U-Net	**91.98**	**91.52**	99.5

The use of manually generated PLFLs as targets is referred to as “Manual-PLFL” and while “Auto-PLFL” refers to the usage of PLFLs generated by our system as training targets.

Best results are shown in [bold].

### CDTL versus GCDT

The effectiveness of the proposed CDT labelling method is entirely dependent on the color characteristics of the targeted dataset. When threshold labels were created using the red soil and green areas of crops, as was the case of the Bean-Field Dataset, there was a limit to how CDT could be used for labeling objects with complex color combinations. In this section, we show how employing the CDT and GCDT methods to generate threshold labels affected the performance of our proposed algorithm. Table 9 illustrates how using a GCDT approach rather than CDTL to generate threshold labels had a negligible effect on the performance of the proposed algorithm.

**Table 9:** The effect of using CDT and GCDT to generate threshold labels for the Bean-Field dataset.

Dataset	Method	mIOU (%)	Precision (%)	Recall (%)	F1-Score (%)
Bean-field	CDT-Labelling	92.43	96.84	95.31	96.07
	GCDT-Labelling	91.36	96.24	94.79	95.51

**Figure 15:**
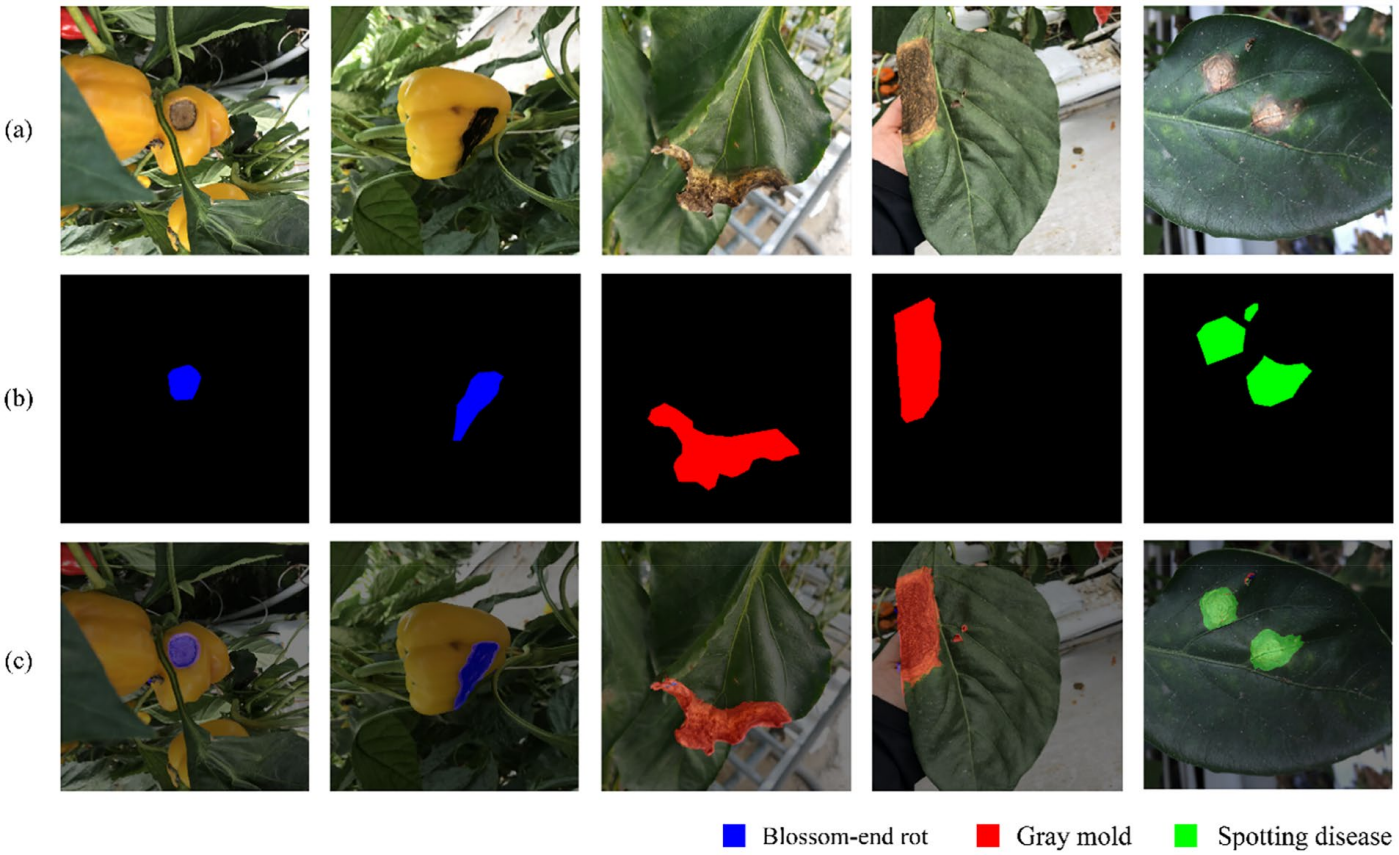
Qualitative results of proposed algorithm on Paprika Disease Dataset. (**a**) input image, (**b**) manual label, and (**c**) overlay of the generated label.

To verify the effectiveness of the GCDT algorithm on a dataset with multiple categories, we performed an experiment using the Paprika-Disease dataset. The qualitative results (Fig. 15) confirm that our proposed algorithm accurately identified several diseases and generated appropriate PLFLs in response. The quantitative results of our proposed algorithm when applied to the Paprika-Disease dataset are provided in Table 10.

**Table 10:** Quantitative Results on the Paprika-Disease dataset having multiple classes.

Dataset	mIOU (%)	IOU			Precision (%)	Recall (%)	F1-Score (%)
		Blossom end rot	Spotting Disease	Gray mold			
Paprika-disease	81.20	76.81	85.54	81.26	95.28	85.25	89.98

## Future works

**Figure 16:**
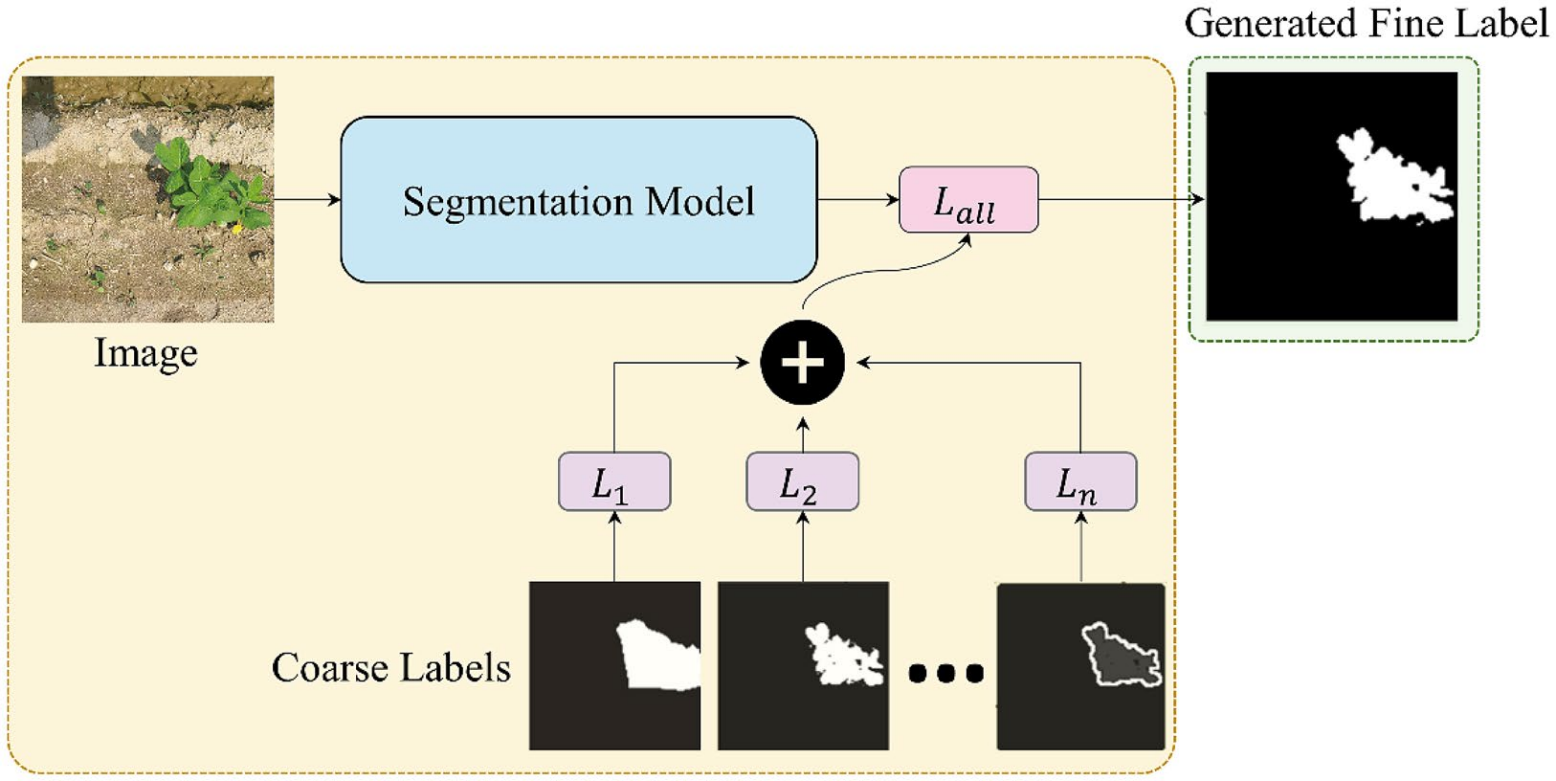
Multi-target single output (MTSO) pipeline of proposed algorithm.

The concurrent use of CLPs (i.e., MGRLs and CDTLs) improved network performance, as seen in Table 6. Future researchers may wish to combine a variety of other automatically generated coarse labels to be used for CNN training. Figure 16 shows how the suggested framework could be easily extended to such coarse label combinations by virtue of its multi-target single output (MTSO) pipeline. In cases of multi-class segmentation, each category in a dataset can have distinct visual and contextual properties, suggesting that the employment of diverse methods of creating coarse labels may improve performance.

## Conclusion

In this study we proposed a method for efficiently and precisely producing segmentation labels using pairs of complimentary coarse labels. MGRL and CDTL made up each corresponding coarse label pair. PLFLs were created using both coarse and fine labels during CNN training. The proposed algorithm was evaluated using three newly constructed datasets (Circle, Bean-Field and Paprika-Disease) as well as two publicly available datasets (CVPPP and CWFID). We also presented a generalization strategy for our proposed CDTL method, which would allow the algorithm to be applied to datasets possessing multiple object classes and complex color distributions.

We compared the performance of our algorithm to that of other state-of-the-art semi- and weakly-supervised segmentation algorithms. Our algorithm outperformed its comparators on both the newly constructed and the publicly available datasets. The segmentation masks generated by a CNN trained using our proposed method achieved a similarity score of over 99% against the segmentation masks generated by a CNN trained using traditional methods. In sum, the proposed labeling strategy considerably minimized the time, cost, and manual labor of fine label production, and its adoption would allow the research community to devote additional resources and time to the creation of new and improved segmentation algorithms.

## Data Availability

The datasets generated and analyzed during the current study can be made available on reasonable request to the corresponding author.

## Code availability

The code is available at https://github.com/hololee/coarse-to-fine-segmentation-labelling.
